# Advanced Applications in Pediatric Dentistry: A Worldwide Perspective of the Last 13 Years

**DOI:** 10.3390/children10101678

**Published:** 2023-10-12

**Authors:** Antonino Lo Giudice

**Affiliations:** Department of General Surgery and Medical-Surgical Specialties, School of Dentistry, Unit of Orthodontics, University of Catania, Policlinico Universitario “Gaspare Rodolico-San Marco”, Via Santa Sofia 78, 95123 Catania, Italy; antonino.logiudice@unict.it

The enhancement of the clinical management of growing patients has always been a great challenge for orthodontists and pediatric dentists. In this regard, pediatric orthodontics and dentistry aim to provide complete preventative and therapeutic care for children and teenagers.

New diagnostic and therapeutic tools are available to enhance the diagnosis and treatment plan strategies in various aspects of oral healthcare for children, including diagnosis, treatment, and consulting expertise for newborns, children and teenagers of all ages, and special care needs. These advancements aim to enhance the quality of care, improve patient experience, and promote better oral health outcomes. Also, they reflect the ongoing commitment to improving the oral health and overall well-being of children, with a focus on minimizing discomfort, anxiety, and long-term dental issues. In this regard, pediatric dentists continue to explore innovative approaches to make dental visits more child-friendly and effective. Below, we provide a brief description of the main clinical topics that underwent significant updating in recent years.

***Digital Radiography and Imaging.*** Digital radiography has become a standard tool in pediatric dentistry. It reduces radiation exposure, provides instant images for diagnosis, and allows for better visualization of dental structures. In pediatric dentistry, digital radiography minimizes radiation risks to developing tissues while providing immediate image results for assessing dental and skeletal development, carious lesions, and traumatic injuries. Three-dimensional imaging techniques, such as cone-beam computed tomography (CBCT), offer precise views of dental and craniofacial structures, aiding in treatment planning for complex cases [1,2].

***Laser Dentistry.*** Laser technology has gained popularity in pediatric dentistry due to its minimally invasive nature and reduced discomfort. Lasers are used for procedures like cavity preparation, soft tissue surgeries, and frenectomies [3,4]. They offer a more comfortable experience for children and can minimize anxiety associated with traditional dental drills [5].

***Silver Diamine Fluoride (SDF).*** SDF is a minimally invasive treatment for dental caries [6]. It involves applying a liquid solution containing silver and fluoride to stop the progression of cavities. SDF can be especially beneficial for young children who may have difficulty sitting through traditional restorative procedures [7].

***Teledentistry.*** The integration of telehealth in pediatric dentistry allows for remote consultations, follow-ups, and monitoring of oral health [8]. This approach is particularly valuable for underserved populations and in situations where in-person visits may be challenging [9].

***Behavioral Management Techniques.*** Pediatric dentists have refined their behavioral management techniques to create a positive and less stressful experience for children. This includes techniques like tell–show–do, distraction, and desensitization, as well as the use of child-friendly language and communication [10,11].

***Pulpotomy Techniques.*** Advances in materials and techniques have improved the success rates of pulpotomies in primary teeth, preserving the tooth’s function and structure [12]. This is particularly important in pediatric dentistry, where the focus is on maintaining primary teeth until they naturally exfoliate [13,14].

***Sedation and Anesthesia***. Pediatric dentists now have a wider range of sedation and anesthesia options to ensure patient comfort and safety during procedures [15]. This includes conscious sedation, deep sedation, and general anesthesia, tailored to the specific needs of each child [16].

***Interceptive Orthodontics.*** Early orthodontic interventions, known as interceptive orthodontics, have gained prominence in pediatric dentistry. Identifying and addressing orthodontic issues in primary dentition can help prevent more severe problems later in life. In this regard, the usage of new materials and devices such as eruption guides or elastodontic devices is becoming more widespread among clinicians for the management of early signs of malocclusion [17,18].

***Preventive Care and Education.*** Pediatric dentists increasingly emphasize preventive care and education. This includes promoting proper oral hygiene practices, dietary guidance, and the use of sealants and fluoride treatments to reduce the risk of cavities [19,20,21,22].

***Customized Pediatric Dental Materials and CAD-CAM fabrication.*** Advancements in dental materials have led to the development of age-specific and pediatric-friendly restorative materials that mimic the natural tooth’s appearance and function. These materials provide better aesthetic outcomes and durability. In pediatric dentistry, this technology enables the fabrication of pediatric-specific restorations that fit accurately and aesthetically, reducing chair time and ensuring optimal clinical outcomes. It can also be used to produce customized orthodontic or functional appliances and, compared to the conventional analogical systems, CAD-CAM devices are digitally designed and produced after careful planning supported by a three-dimensional evaluation of the patients’ characteristics [23,24].

Despite the increasing interest in pediatric dentistry updates, there is still the necessity of future research investigating the effectiveness of new clinical protocols for children. In this regard, the readers of this Special Issue entitled “New Clinical Evidence in Pediatric Orthodontics and Pediatric Dentistry” will be presented with new insights into this new cutting-edge topic in pediatric dentistry.
